# Longevity

**Published:** 1906-08-18

**Authors:** 


### Longevity.

It was thought in uncritical times that the life of man might be extended to a hundred and fifty years and upwards. Then came a reaction when Mr. Thoms denied the existence of centenarians except in the rarest instances. The labours of Sir George Murray Humphry put the question on a scientific basis and proved that there is about one centenarian to every 127,000 people, and that of seventy authenticated cases no one reached 110 years, three only are said to have been 108, and one 106. The records of centenarians show that the full exercise of the various powers, mental and bodily, is conducive to great age, so that there need be no fear of entering heartily, actively, and with full interest and energy into the assigned work of life, physical or mental. Work enjoyed as it should be promotes health of body and especially, if stimulated by other motives than personal ambition and gain, engenders that cheerful and placid frame of mind which is one of the adjuncts of centenarianism. The inhabitants of any countryside, as in Delabole in North Cornwall, point with pride to the number of hale and hearty octogenarians, nonagenarians, and centenarians living among them as an evidence of their healthy environment and hygienic lives. So in Paris, with its 10,509 octogenarians, 620 nonagenarians, 89 of whom are approaching their hundredth year. Six inhabitants of Paris are more than 102 years of age.

But if centenarianism is rare amongst human beings there are animals with a much more extended term of life. The giant tortoise at Mauritius is known to have lived in captivity in the Court House Garden since 1764, so that it has been in existence for at least 150 years. "Drake," the venerable tortoise which has lately died at the Zoological Gardens, was supposed to be still older. It was captured in the Galapagos Islands towards the end of the eighteenth century, and was brought to England eighty-five years ago. After several changes of ownership he finally found comfortable quarters for his declining years in the Zoological Gardens. His exact age is unknown, but at the time of his capture he appeared to have a half-effaced date cut on his shell of which the first two figures were 16, so that it is possible he may have lived in three centuries. Carp, crocodiles, ravens, and elephants are known on excellent authority to attain to a good old age, but it is impossible to tell even approximately the natural terms of life of animals living under natural conditions. If it were possible to know the age attained by the sperm whale or the great rorqual we might guess at the age or rate of growth of some of the enormous extinct animals because, large as they were, they were small in comparison with modern cetacea. Longevity in no way depends upon size, and the normal life of an animal is taken roughly as being three times the length of time it takes to attain maturity. In the far-off ages when the dinosaurs and the other gigantic reptiles flourished, warmth, protection and a plentiful supply of suitable food may have led to rapid growth without undue prolongation of years. There is evidence that the same great climatic change put a summary end to the existence of the creatures, for they passed away absolutely, leaving no descendants either changed or unchanged. But recent discoveries have shown that some animals supposed to have been extinct for thousands of years have lived on into recent times. The remains of many individuals of the Mylodon or gigantic ground sloths, which were as big as bulls, have been found in a cavern on the fiord of the Ultima Speranza in Southern Patagonia, and not only the bones with blood, tendons, and cartilages, but even the skin and dung. The bones have been broken by men to get at the marrow, so that it is clear that quite recently men have herded and eaten animals supposed to have become extinct in prehistoric times.

